# Nonwoven Materials Produced by Melt Electrospinning of Polypropylene Filled with Calcium Carbonate

**DOI:** 10.3390/polym12122981

**Published:** 2020-12-14

**Authors:** Sergey N. Malakhov, Petr V. Dmitryakov, Evgeny B. Pichkur, Sergey N. Chvalun

**Affiliations:** National Research Centre “Kurchatov Institute”, Akademika Kurchatova pl., Moscow 123182, Russia; dmitryakov@mail.ru (P.V.D.); eugene.pichkur@gmail.com (E.B.P.); s-chvalun@yandex.ru (S.N.C.)

**Keywords:** melt electrospinning, nonwovens, composites, polypropylene, calcium carbonate

## Abstract

Nowadays, polypropylene-based nonwovens are used in many areas, from filtration to medicine. One of the methods for obtaining such materials is melt electrospinning. In some cases, it is especially interesting to produce composite fibers with a high degree of filling. In this work, the influence of the filling degree of isotactic polypropylene with calcium carbonate on the structure and properties of nonwoven materials obtained by melt electrospinning was studied. It was shown that electrospinning is possible, even at a filler content of 50%, while the average diameter of the fibers increases with the growth in the content of calcium carbonate. The addition of sodium stearate significantly reduces the diameter of the fibers (from 10–65 to 2–10 microns) due to reducing viscosity and increasing the electrical conductivity of the melt. Wide-angle X-ray diffraction analysis and IR spectroscopy reveal that the initial polymer and composites are characterized by the presence of stable α-form crystals, while nonwovens show a predominance of smectic mesophase. The addition of calcium carbonate leads to an increase in the hydrophobicity of the composite films, the addition of sodium stearate results in a decrease of hydrophobicity, while all nonwovens demonstrate superhydrophobic properties.

## 1. Introduction

Polypropylene is a commodity polymer that combines a number of useful properties, i.e., low density, low moisture absorption, mechanical strength, high chemical resistance, and low cost. Fibers and nonwovens based on polypropylene are of considerable practical interest, e.g., in the fields of filtration [1,2,3], oil sorption and oil–water separation [4], protective coatings [5] and sensors [6], as well as in medicine and tissue engineering [7,8]. The average diameter of fibers in these materials depends on the production parameters and can vary from a few to dozens of micrometers. A number of methods can be used to obtain such materials, but due to the limited solubility of polypropylene, its processing into a fiber from a solution is difficult and, as a result, it is an object of primarily research interest. In this regard, there are a few works devoted to electrospinning from polypropylene solutions: it is reported that nonwovens were produced from solutions in decalin [9] or from mixtures of solvents based on decalin [10] and cyclohexane [11], while heating the solution to 70–130 °C was required to dissolve the polymer. On the other hand, it is possible to produce fibrous polypropylene materials from the melt by electrospinning [2,3,4,5,7,8,9,12,13,14], melt-blowing [1,15], centrifugal spinning [16] or by combining several of these methods [17]. Moreover, nonwovens can be produced, not only from polypropylene homopolymers, but also from its copolymers [18] and blends with other polymers [19,20,21].

Currently, polymer composites are the object of close attention from both scientific and practical points of view. The addition of a small amount of filler into a polymer matrix can significantly improve mechanical, electrophysical, thermal, barrier characteristics of the material or give it new properties, e.g., antibacterial or wound healing. Thus, layered silicate fillers (such as montmorillonite) improve the mechanical and thermal properties of the polymer [22]; hydroxyapatite can accelerate the growth of bone tissue [23]; silver nanoparticles provide the material bactericidal properties [24]; titanium dioxide has high photocatalytic activity, which is useful for air and water cleaning [25]; and the addition of carbon nanotubes allows to obtain electrically conductive polymer composites [26]. At the same time, the filler content in these cases is small and varies from fractions to a few percent. A filler can also be used to reduce the cost of manufactured products. In this case, the filler content can reach 50–60%, and it should be inexpensive. One of the most common fillers of this kind is calcium carbonate [27]. Filling polypropylene with calcium carbonate allows to further reduce the price of the material [28,29,30,31,32,33,34,35]. Thus, highly filled composite nonwoven materials based on polypropylene are of practical interest, e.g., for filtration and sorption purposes. However, as of now nonwovens are obtained from pure polymer or with low filler content (up to 5%).

One of the factors limiting the usage of the melt electrospinning method is the higher average diameter of the fibers due to the higher viscosity of the polymer melts compared to the polymer solutions. Moreover, the introduction of a mineral filler usually leads to an additional increase in viscosity, and as a result, an increase in the diameter of the fibers [36]. To reduce the melt viscosity, one can increase the temperature [13] or use the polymers of lower molecular weight [8] or introduce commercially available low-molecular-weight additives for polyolefins [37]. In addition, we have previously shown that addition of higher fatty acid salts allows to reduce viscosity and enhance the electrical conductivity of the melts [14].

Thus, the aim of this work is to determine the effect of the high filler content in polypropylene on the possibility of generating fibers by melt electrospinning, as well as to study the morphology, structure, and properties of the obtained nonwoven materials.

## 2. Materials and Methods 

### 2.1. Materials

Polypropylene (PP) Balen 01,270 with a melt flow index of 25.6 purchased from Ufaorsintez (Ufa, Russia) and calcium carbonate (CC) Calmast CM360 (average particle size 0.8 μm) was used. Sodium stearate (SS) was produced by Panreac (Barcelona, Spain) and used (at a concentration of 3 wt.%) to improve viscosity and electrical conductivity of the melts, as described previously [14,38]. All reagents were used as received, without any further purification. Composites for electrospinning were prepared by mixing the components in a twin-screw extruder HAAKE PolyLab (Thermo Fisher Scientific, Waltham, MA, USA) at 180 °C, and then re-granulated

### 2.2. Electrospinning

**Figure 1:**
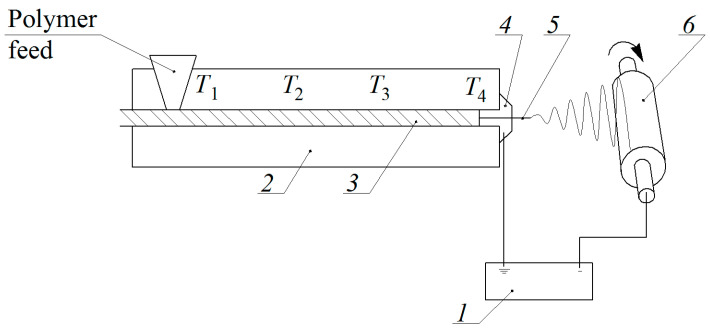
Experimental setup for melt electrospinning: 1—high voltage supply, 2—feeding device (extruder), 3—screw, 4—nozzle, 5—fiber, 6—collecting device.

An experimental setup based on a single screw extruder Plasti-Corder PLE-330 (Brabender GmbH & Co. KG, Duisburg, Germany) with four heating zones (Figure 1) was used for melt electrospinning. Pellets of the previously prepared composite were loaded into an extruder (2), where it was melted, and then the melt was forced by a screw (3) to the nozzle. Passing through the nozzle (4), the melt formed a primary jet (5), which entered into an electric field created by a high-voltage source (1). Under the action of the electric field, the primary jet was drawn and split many times and drifted to the collecting electrode (6), forming a nonwoven material ready for further use. To prevent premature degradation of the polymer, the temperature of the first zone (T_1_) was set at 170 °C, the temperature of the second and the third zones (T_2_-T_3_) was set at 200 °C. Electrospinning was performed at a nozzle temperature (T_4_) of 330 °C. The extruder was grounded and the high voltage (135 kV) generated by the Spellman SL130PN30 source (Spellman High Voltage Electronics Corporation, Hauppauge, NY, USA) was applied to the cylindrical drum (collecting electrode) equipped with an electric motor drive. The diameter and width of the drum were 15 and 25 cm, respectively; the rotation speed was 1.5–2 rpm and the distance between the nozzle and the collector was 45 cm.

### 2.3. Characterization

Micrographs of the nonwoven materials were taken with a Axio Imager.M2m optical microscope (ZEISS, Oberkochen, Germany) and an Versa 3D (Thermo Fisher Scientific, Waltham, MA, USA) scanning electron microscope at an accelerating voltage of 5 kV without any conductive coatings. Image processing and analysis of fiber diameters were carried out using ImageJ 1.49v software.

The melt viscosity was determined using a CEAST Smart Rheo 2000 (Instron, Norwood, MA, USA) capillary rheometer (capillary length 40 mm, channel diameter 1 mm) at a temperature of 230 °C and shear rates of 100–3000 s^–1^. To study the electrical conductivity of the materials, a Novocontrol Concept 40 broadband dielectric spectrometer (Novocontrol Technologies, Montabaur, Germany) equipped with an Alpha-A active sample cell was used. The measurements were made at a temperature of 150 °C, voltage of 1 V, and frequency of 1 Hz. The samples for the electrical conductivity and contact angles measurements were made with a HAAKE MiniJet II (Thermo Fisher Scientific, Waltham, MA, USA) laboratory molding machine. The temperature of the polymer melt was 200 °C, the temperature of the mold 75 °C, injection and post-process pressures 550 and 200 bar, time 30 and 20 s respectively. A disk-shaped mold with a diameter of 25 mm and a thickness of 1.5 mm was used.

Thermal gravimetric analysis was performed with a Pyris 1 TGA (PerkinElmer, Waltham, MA, USA) in the temperature interval 50–550 °C in a nitrogen flow (100 mL/min). The sample weight was about 10 mg for pellets and 5 mg for nonwoven materials. IR spectra were recorded with a Nicolet iS5 (Thermo Fisher Scientific, Waltham, MA, USA) spectrometer with an iD5 ATR accessory in the range of 550–4000 cm^−1^ with an averaging of 32 scans. X-ray diffraction (XRD) analysis of the samples was performed on a Rigaku SmartLab (Rigaku Corporation, Tokyo, Japan) diffractometer (CuK_α_-radiation, λ = 1.5408 Å). Contact angles of the nonwoven materials were measured using a DSA30E drop shape analyzer (KRÜSS GmbH, Hamburg, Germany). The liquid phase was water and the droplet volume was 5 μL.

## 3. Results

### 3.1. Viscosity and Conductivity

**Figure 2:**
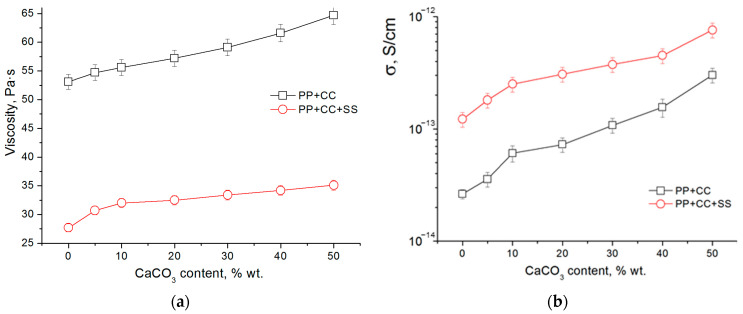
Effect of CaCO_3_ content on the viscosities (**a**) and conductivities (**b**) of polypropylene composites.

As has been previously reported in studies on the electrospinning of polymer melts, viscosity and electrical conductivity are key parameters that determine the characteristics of the resulting fibers [39]. The effect of the filler content on the viscosity is shown in Figure 2a. Melts of both pure and filled polypropylene demonstrate a non-Newtonian flow pattern, while their viscosity monotonically increases with the increase in the content of calcium carbonate in the composite, which is in good agreement with the published data [31,32]. The addition of sodium stearate reduces the viscosity of the melt by about half, which should simplify the generation of thin composite fibers.

Figure 2b shows the results of a study of the electrical conductivity of polypropylene samples with different degrees of filling. As one can see from the obtained data, the specific electrical conductivity of composites increases as the filler content increases. However, the values of specific electrical conductivity are relatively low—in the range of 10^−14^–10^−13^ S/cm. The addition of sodium stearate can further increase the conductivity of the material. This effect is caused by the appearance of additional charge carriers, also, their mobility increases with increasing temperature. These factors lead to an increase in electrical conductivity for both polar [39], and non-polar polymers [14].

Thus, the addition of calcium carbonate has a dual effect on the characteristics of the polypropylene melt in relation to the electrospinning process. On the one hand, an increase in electrical conductivity should promote the formation of thinner fibers; on the other hand, an increase in viscosity leads to an increase in the diameter of the producing fibers.

### 3.2. Fiber Properties

**Figure 3:**
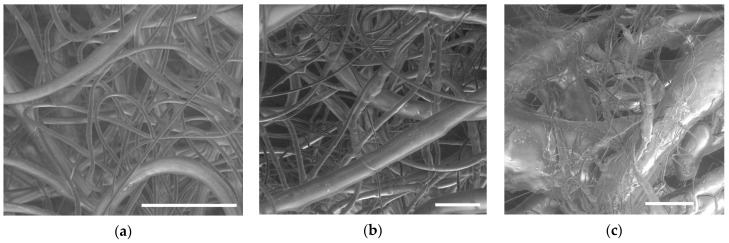
Micrographs of the nonwoven materials: PP+SS (**a**), (90PP+10CC)+SS (**b**), (50PP+50CC)+SS (**c**). Scale bar is 40 μm.

**Figure 4:**
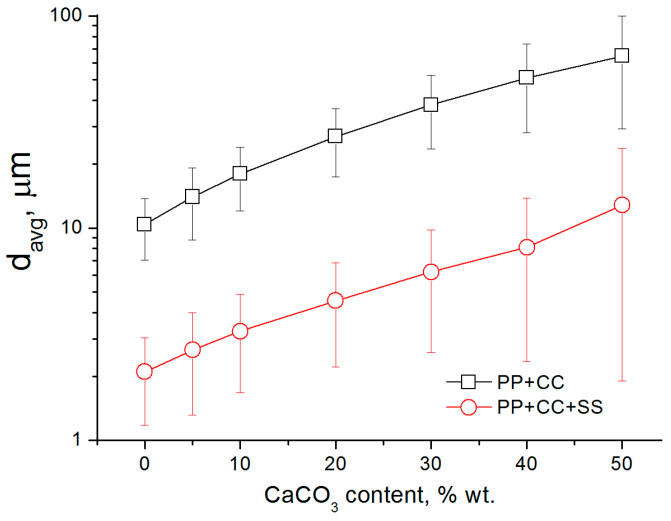
Effect of CaCO_3_ content on the average fiber diameters.

The process of electrospinning polypropylene starts only at ~280 °C, while polyamide-6, despite its higher viscosity, is capable of generating fibers at 255 °C [39]. Thus, it can be concluded that the factor limiting the spinnability of polypropylene at lower temperatures is insufficient electrical conductivity, which is significantly lower than that of polyamide. As the temperature increases, the average diameter of the obtained fibers decreases, and at 330 °C it is about 10.4 µm. The addition of sodium stearate allowed to reduce the average fiber diameter to 2.1 µm (Figure 3a) by decreasing the viscosity and increasing the electrical conductivity of the melt. The resulting fibers have a round shape and a smooth surface while a relatively wide fiber diameter distribution is observed. The average diameter of the resulting fibers can be controlled within a fairly wide range by regulating the content of sodium stearate [14]. In the case of melt electrospinning of the composites, the average diameter of the fibers increases as the content of calcium carbonate in the sample grows (Figure 4). In addition, the composite fibers have a non-circular shape and a complex surface morphology (Figure 3b). For the fibers with a high filling degree (40–50%) the distribution of fibers by diameter is close to bimodal due to an increase in the content of thick fibers. A number of defects are observed such as spherical and spindle-shaped thickenings and microdroplets (Figure 3c). It should be noted that in this case a certain number of unstretched polymer droplets are observed at the edges of the collecting drum and around it. This fact can be explained by the size distribution of the calcium carbonate particles. It can be expected that electrospinning produces better results with the use of nano-sized fillers.

Soaking the composite materials in hydrochloric acid results in the extraction of only a small amount of filler. Even with a calcium carbonate content of 50 wt.%, the total mass loss of the fibrous sample is no more than 13%, i.e., 26% of the filler content. This indicates that most of the calcium carbonate is covered by a polymer. SEM images of the samples after soaking in HCl are shown in Figure S1. As one can see, the soaking of the nonwovens in hydrochloric acid does not result in formation of complex porous fibers morphology.

### 3.3. Structural Analysis

**Figure 5:**
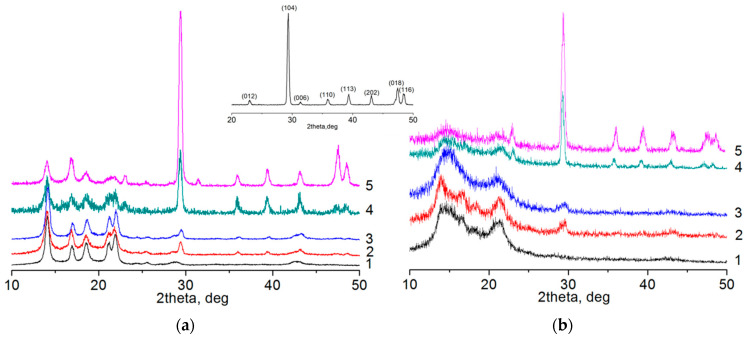
XRD patterns of films (**a**) and nonwovens (**b**) from pure PP (1) and composites of 95PP+5CC (2), (95PP+5CC)+SS (3), (70PP+30CC)+SS (4), (50PP+50CC)+SS (5). Inset—pattern of CaCO_3_ powder.

It can be expected that the addition of filler will affect the structure of the polymer. That is why the supramolecular structure of the samples was characterized by wide-angle X-ray diffraction. The X-ray patterns for composites are presented in Figure 5a and for nonwovens are shown in Figure 5b. According to X-ray data, the initial polypropylene and composites based on it are characterized by the presence of the reflections (110) at 14.1°, (040) at 16.9°, (130) at 18.5°, (111) at 21.2°, (131) + (041) at 21.9°, (060) at 25.3°, and (220) at 28.4°, corresponding to a stable α-form with a monoclinic crystal lattice [9,40]. Reflections corresponding to the β- or γ-form (e.g., (300) and (117), respectively) are not observed. In addition, calcite reflections appear in samples containing calcium carbonate: (104) at 29.4°, (113) at 39.4°, (202) at 43.1°, (018) at 47.4°, and (116) at 48.4° [41]. An increase in the filler content leads to an increase in the intensity of these reflections.

During the melt electrospinning process, the polymer jet has a very short cooling time from a temperature above 300 °C to room temperature, while it is simultaneously drawn by a strong electric field (with a linear strength of about 3 kV/cm). As a result, the crystal structure of the polymer undergoes significant changes (Figure 5b) associated with the appearance of a low-ordered smectic mesophase, which is characterized by the presence of two broad diffuse reflections on the diffraction patterns of materials. At the same time, in thicker fibers (e.g., from pure polypropylene and with the addition of calcium carbonate but without sodium stearate), where the polymer is cooled more slowly, the formation of a certain number of α-form crystallites is observed, and in thinner fibers only mesophase is observed. A similar effect is observed in the case of rapid crystallization (quenching) of polypropylene thin films [40]. A comparative analysis of the obtained results showed that only mesophase is observed at a fiber diameter of up to 8–9 µm. If the fiber diameter is higher, then crystallites of a more equilibrium α-phase are also formed. Its content grows as the fiber diameter increases. In addition, all typical calcium carbonate reflections are observed on X-ray patterns of nonwoven composite materials, the intensity of which increases as the content of filler increases.

**Figure 6:**
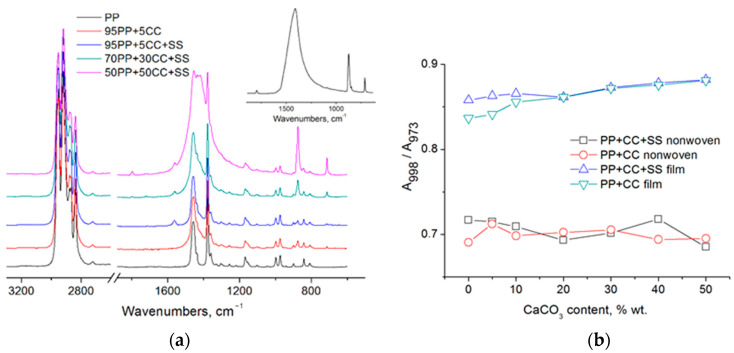
FTIR spectra of the produced nonwoven materials (**a**); effect of CaCO_3_ content on the A_998_/A_973_ ratio (**b**). Inset—spectra of pure calcium carbonate.

The study of samples by IR spectroscopy revealed (Figure 6a), that the spectra of all the samples contain antisymmetric and symmetric stretching vibrations of C–H in methyl (2953 and 2872 cm^−1^, respectively) and methylene (2919 and 2840 cm^−1^, respectively) groups; bending vibrations of C–H in methyl groups: antisymmetric (1450 cm^−1^) and symmetric (1378 cm^−1^); bending vibrations of C–H bonds in methylene groups: scissoring (1457 cm^−1^) and wagging (1304 cm^−1^) [7,8]. Bands 1219, 1167, 1101, 998, 973, 941, 899, 841, and 808 cm^−1^ in isotactic polypropylene are called crystallinity bands and belong to isotactic chains of various lengths [42]. In composites and fibers containing a filler, bands appear in the region of 712, 874, and 1410 cm^−1^, corresponding to vibrations of calcium carbonate in the form of calcite [43]. The intensity of these bands increases as the content of CaCO3 in the sample increases. In addition, a band at ~1560 cm^−1^, which is related to antisymmetric stretching vibrations of the carboxylate anion [14] appears in the IR spectra of materials containing sodium stearate.

It should be noted that depending on the conditions of crystallization of isotactic polypropylene, the ratio of the intensity of the absorption bands at 998 and 973 cm^−1^ changes, which can be used to evaluate the regularity of the polymer chain stacking [44]. The initial polymer is characterized by a ratio of A998/A973 equal to 0.84, in film samples, as the content of calcium carbonate increases, it increases to 0.88, for nonwovens it decreases to 0.69-0.71 (Figure 6b). The obtained data indicate a decrease in the proportion of polymer chains in the helical conformation 31 during electrospinning.

### 3.4. Thermal Properties

**Figure 7:**
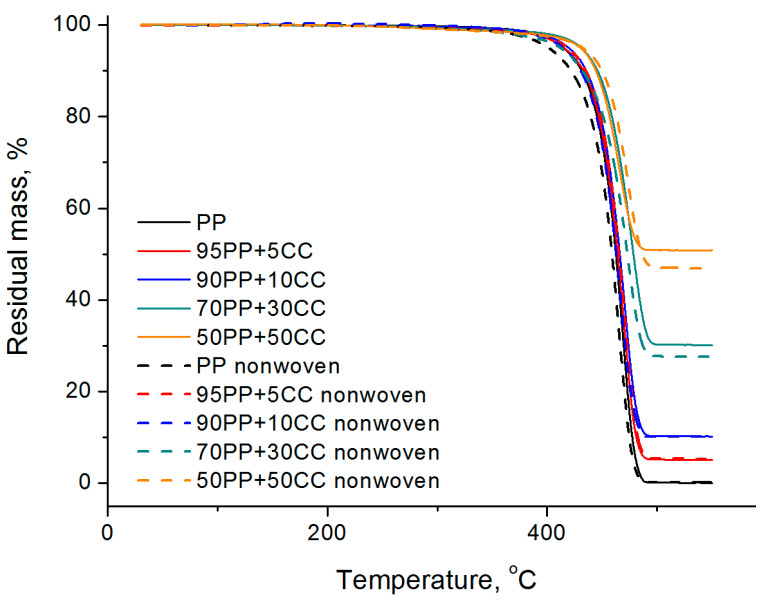
TGA curves of the composites (solid lines) and nonwoven materials (dash lines).

The produced composites as well as nonwovens were studied by thermogravimetric analysis; the resulting curves are shown in Figure 7. As can be seen from the obtained data, the granule and fiber from a pure polymer decompose almost completely (the residual mass is 0.1–0.2%). The samples obtained with the addition of sodium stearate behave similarly (the residual mass is 0.5–1.2%). Polypropylene has very low moisture absorption, resulting in a mass loss only of 0.1–0.2% in the range up to 250 °C for both composites and nonwovens based on it. For comparison, the fibers based on polyamide-6, which is a very hygroscopic polymer, can lose up to 4% of its mass up to 200 °C [36].

For composites, the residual masses correspond to the masses of the calcium carbonate loadings. For composite nonwovens with a percentage of filler up to 20% inclusive, the residual mass also corresponds to the filler content in the original composites. However, electrospinning from composites with a higher filling degree leads to a small decreasing of the residual mass of calcium carbonate in the obtained fiber (up to 28.5–29% for a composite with 30% calcium carbonate, up to 37–38% for a 40% composite and up to 46–47% for a composite with 50% filling degree). A possible explanation of this fact is that droplets observed at the edges of the collecting drum and around it contain an excess of filler and represent the largest fraction of calcium carbonate particles (or their aggregates) covered with a thin layer of the polymer.

The IR spectrum of the dry residue after TGA fully corresponds to the spectrum of pure calcium carbonate (see inset in Figure 6).

### 3.5. Wettability

Polypropylene is well-known as a hydrophobic polymer, so polypropylene nonwovens exhibit hydrophobic and even superhydrophobic properties [9,14].

**Figure 8:**
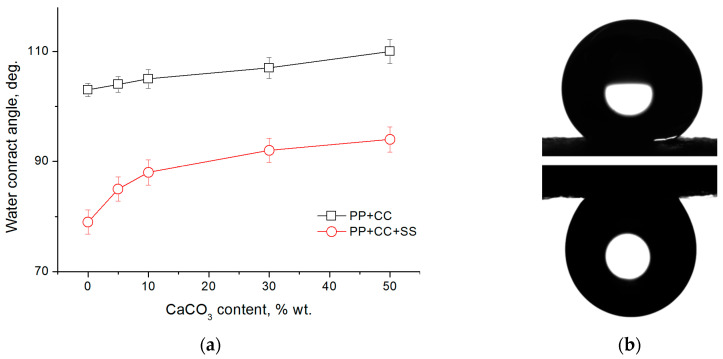
(**a**) Effect of CaCO3 content on the wettability of the polymeric films; (**b**) optical image of a water droplet on the surface of the (90PP+10CC)+SS nonwoven material after 60 seconds from deposition (top) and after rotating the material by 180° (bottom).

Films made of pure polypropylene show an average water contact angle of 103 degrees. The addition of 3% sodium stearate (which is an ionic surfactant) leads to a significant increase in the hydrophilicity of the film: the value of the contact angle decreases to 79 degrees. A similar effect was previously observed for samples based on polyamide-6 [39]. For composite films, the contact angle increases as the content of calcium carbonate in the sample increases (Figure 8a).

However, nonwovens (both made of pure polypropylene and containing surfactants and fillers) show superhydrophobic properties: the values of the water contact angle are in the range of 143–147 degrees (Figure S2) and do not significantly depend on the presence, concentration, and type of additive, and the exact value of the contact angle is determined by the local surface morphology.

If the surfactant is removed from the surface of the film containing sodium stearate by immersing and holding the film in water, the value of the contact angle becomes equal to that of the film from pure polypropylene. In contrast, for nonwoven materials, the value of the contact angle after immersing in water does not change.

It should be noted that individual water droplets applied to the surface of nonwoven material do not roll down when the sample is tilted, but are held on the surface even when the fabric is turned upside-down at 180 degrees (Figure 8b). This effect is similar to the rose petal effect and is due to the morphology of the material surface which has a micro-rough structure: part of the applied droplet falls into the space between the fibers and is securely held there, preventing the movement of the droplet, i.e. wetting occurs according to the Wenzel model [45,46].

## 4. Conclusions

In this work, melt electrospinning of polypropylene filled with calcium carbonate was investigated for the first time. It was shown that polypropylene/CaCO_3_ composites retain the ability to generate fibers from the melt even when the filling degree is up to 50 wt.%. The average diameter of the fibers increases as the content of calcium carbonate in the composite rises. To reduce the average fibers’ diameter, the addition of sodium stearate can be used; it decreases the viscosity and enhances the electrical conductivity of melts. Thus, with the addition of 3 wt.% of the stearate, the average diameter of the fibers is reduced by about five times; for a greater reduction, the concentration of sodium stearate can be increased. At high degrees of filling, the content of thick fibers and the number of their defects grows significantly. Thus, the optimal filling is up to 20–30 wt.%, in this case, such significant changes are not observed. Soaking the material in hydrochloric acid does not result in formation of complex porous fibers morphology, because most of the CaCO_3_ is covered by the polymer.

The study of the supramolecular structure of polypropylene has shown that during electrospinning from the melt, a transition from the stable α-form crystals to the smectic mesophase in nonwoven materials occurs due to rapid cooling of the polymer under high-speed drawing in an electric field. At the same time, the supramolecular structure of the polymer in nonwovens does not depend on the presence of additives but is determined only by the diameter of the obtained fibers. In thick fibers, where the crystallization process is slower, crystallites of a more equilibrium α-phase are formed, while in thinner fibers (up to 8–9 µm in diameter), only a mesophase is formed. Nonwoven materials obtained in this work exhibit superhydrophobic properties and their wetting occurs according to the Wenzel model.

## Supplementary Materials

The following are available online at https://www.mdpi.com/2073-4360/12/12/2981/s1, Figure S1: SEM images of nonwoven materials made from PP/CaCO_3_ composites after soaking in HCl, Figure S2: optical images of water droplet after 60 seconds from deposition on the surface of the nonwoven materials.
