# Supplementary material for: Nonwoven Materials Produced by Melt Electrospinning of Polypropylene Filled with Calcium Carbonate
Source: Polymers (Basel). 2020 Dec 14;12(12):2981. doi: 10.3390/polym12122981 (PMC7764975; doi:10.3390/polym12122981)
Supplement: Supplementary file 1 [file polymers-12-02981-s001.pdf]

Supplementary Material

NONWOVEN MATERIALS PRODUCED BY MELT ELECTROSPINNING OF POLYPROPYLENE FILLED WITH CALCIUM CARBONATE

Sergey N. Malakhov*, Petr V. Dmitryakov, Eugene B. Pichkur, Sergey N. Chvalun

National Research Centre "Kurchatov Institute, Moscow, Russia

*Correspondence: s.malakhov@mail.ru (S.N.M.)

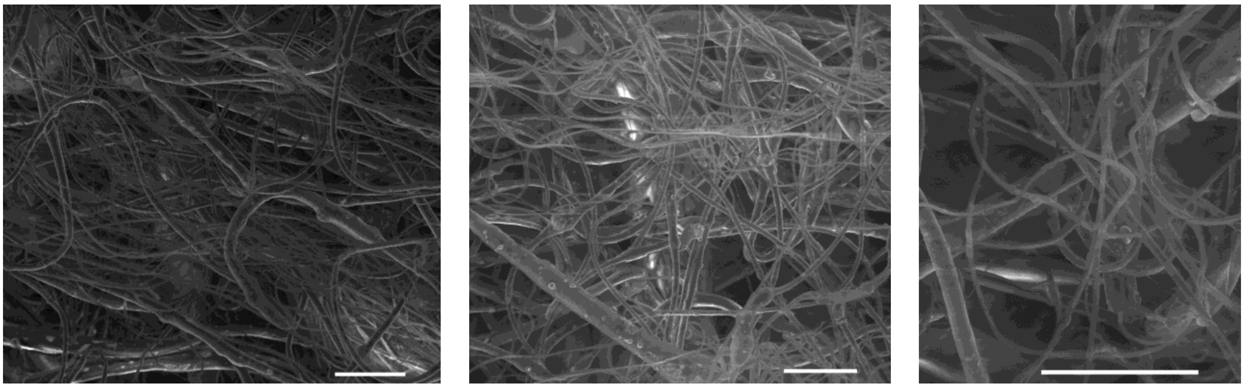

Figure S1. SEM images of nonwoven materials made from PP/$CaCO_3$ composites after soaking in HCl. Scale bar is 50 μm

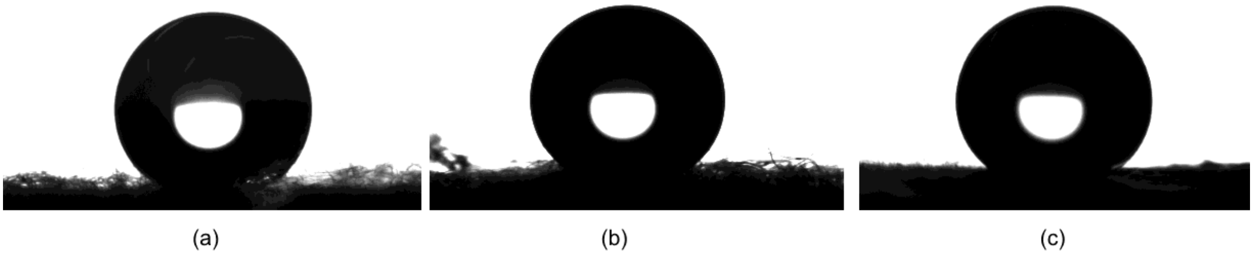

Figure S2. Optical images of water droplet after 60 seconds from deposition on the surface of the nonwoven materials made from pure PP (a), PP + 10% $CaCO_3$ (b), PP + SS (3)
